# NOAA Open Data Dissemination: Petabyte-scale Earth system data in the cloud

**DOI:** 10.1126/sciadv.adh0032

**Published:** 2023-09-20

**Authors:** Denis S. Willett, Jonathan Brannock, Jenny Dissen, Patrick Keown, Katelyn Szura, Otis B. Brown, Adrienne Simonson

**Affiliations:** ^1^ Cooperative Institute for Satellite Earth Systems Studies (CISESS), North Carolina Institute for Climate Studies, North Carolina State University, Asheville, NC, USA.; ^2^ NOAA Open Data Dissemination (NODD), National Oceanic and Atmospheric Administration, Asheville, NC, USA.

## Abstract

NOAA Open Data Dissemination (NODD) makes NOAA environmental data publicly and freely available on Amazon Web Services (AWS), Microsoft Azure (Azure), and Google Cloud Platform (GCP). These data can be accessed by anyone with an internet connection and span key datasets across the Earth system including satellite imagery, radar, weather models and observations, ocean databases, and climate data records. Since its inception, NODD has grown to provide public access to more than 24 PB of NOAA data and can support billions of requests and petabytes of access daily. Stakeholders routinely access more than 5 PB of NODD data every month. NODD continues to grow to support open petabyte-scale Earth system data science in the cloud by onboarding additional NOAA data and exploring performant data formats. Here, we document how this program works with a focus on provenance, key datasets, and use. We also highlight how to access these data with the goal of accelerating use of NOAA resources in the cloud.

## Teaser

The NOAA Open Data Dissemination program makes petabytes of NOAA environmental data publicly and freely available on the cloud.

## INTRODUCTION

The National Oceanic and Atmospheric Administration (NOAA) Open Data Dissemination (NODD) program makes NOAA environmental data publicly and freely available on Amazon Web Services (AWS), Microsoft Azure (Azure), and Google Cloud Platform (GCP). NOAA generates terabytes (TB) of data per day from its many observing and sensing platforms including satellites, sensors, models, and forecasts. These data inform every aspect of the Earth system from the atmosphere to the oceans and are used by the government, researchers, and private industry to understand everything from the dynamics of natural disasters to climate change.

NODD began as an experimental collaboration in 2015 to explore the technical, economic, and financial means by which NOAA data could be made publicly available to a wider audience using emerging cloud technologies (*1*). Since 2015, NODD has expanded considerably and is now a NOAA enterprise dissemination program. As a dissemination platform that leverages cloud technologies for improved data accessibility, NODD is not a data archive. Other NOAA programs, like the National Centers for Environmental Information (https://ncei.noaa.gov/), have archiving responsibilities, in addition to data dissemination responsibilities.

NODD is supported by collaborative multiyear agreements with AWS, Azure, and GCP, which have made a commitment to maintain open NOAA data on the cloud free of charge to NOAA and to data users. The development of this program has highlighted the value and utility of hosting open data in the cloud to the cloud service providers (AWS, Azure, and GCP), solution providers, end users, and other stakeholders who use and rely on these data.

NODD fulfills many of the Mandates of the Foundations for Evidence-Based Policymaking Act of 2018 (H.R. 4174) or Evidence Act, particularly the requirements set forth in Title II: The Open Government Data Act, where government data are open by default. Specifically, NODD makes NOAA data assets publicly available in open formats, publishes public government data assets as machine-readable data, maintains a data inventory, engages the public in using the data assets of the agency, and provides the public with the opportunity to request specific data assets. Also, in accordance with the Evidence Act, NODD monitors information on the usage of such assets to facilitate prioritization and improvement in NOAA public data offerings. This monitoring is at a high level and includes total storage, data accessions in terms of the total amount of bytes used, and interactions or requests for the data. Because of the completely open nature of NODD data and support for anonymous access, there is a fundamental privacy limitation on individual use metrics.

NODD now supports a number of key operational NOAA datasets including Next Generation Weather Radar, Geostationary Operational Environmental Satellites (GOES), and Global Forecast System (GFS), among many others (see: https://noaa.gov/nodd/datasets). The availability of these datasets on the cloud is driven by demand; users, stakeholders, and cloud service providers collaborate with NODD to identify and prioritize high-value, high-impact datasets for immediate use. These datasets are then moved to the cloud using a combination of NODD-managed and, in some cases, data-owner–managed pipelines. Access to these datasets is then supported through the platforms of each cloud service provider, which enables extremely performant, parallel access to the data holding. Performant here is taken to mean low latency and scalable. Performant access means that the data are available without waiting long periods of time to access them (low latency) and all of the data needed, whether it be terabytes or petabytes, are accessible at the same time and/or can be accessed frequently (scalable).

The nature of the NODD agreement with each cloud service provider makes this type of access possible. These agreements were designed with longevity and permanence in mind to create a win-win-win public-private partnership that benefits the cloud service provider, NOAA, and the data user. In addition to long-term, low-cost contracts that allow NOAA to allocate certain datasets, each cloud service provider has requested making additional data available. Each cloud service provider benefits, and is incentivized to continue the program, because data available on their platform are easier to analyze via data-adjacent paid cloud computing resources. NOAA benefits from a highly performant and accessible data dissemination platform. The end data user benefits from extremely performant high-volume, low-latency access to environmental data.

**Fig. 1.**
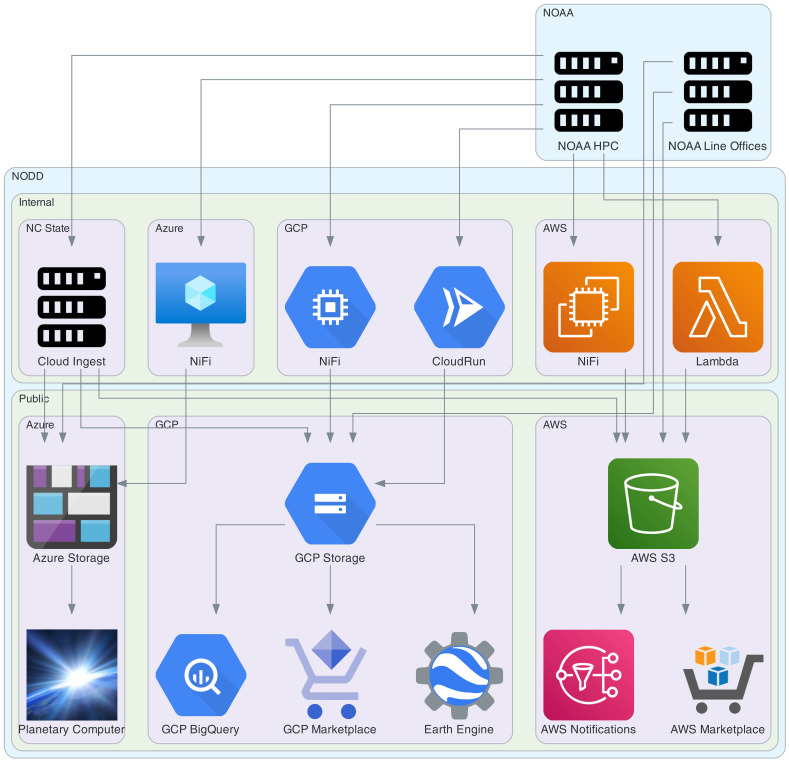
NODD pipelines. NODD data originate in NOAA systems and at NOAA Line Offices and then move to the cloud either through Apache NiFi instances running on each of AWS, Azure, and GCP, through the Cloud Ingest Pipeline developed by the Cooperative Institute for Satellite Earth System Studies at North Carolina State University through the North Carolina Institute for Climate Studies, or directly to cloud object stores from internal NOAA systems. NODD data in cloud object stores (AWS S3, Azure Storage, and GCP Storage) are publicly and freely available. NODD data and notifications can also be accessed through additional publicly and freely available cloud services, for example, Planetary Computer, Earth Engine, and AWS Marketplace (bottom row of services in figure).

A tremendous amount of work and investment was applied to developing the infrastructure (Fig. 1) to support open access to NOAA data via the cloud. Because of this investment and infrastructure, data are provided openly and freely. Anyone with an internet connection can access these data without a subscription, registration, or payment of any kind. Free access (and egress) democratizes access to environmental data about the Earth system, encourages open science, and facilitates participation and utility across economic sectors.

Data are provided in cloud-based object storage. This storage allows for massively parallel, simultaneous access without throttling and provides orders of magnitude increased performance compared to traditional FTP or HTTP sites providing data products. Some data are provided in formats that further increase performance, allowing for on-the-cloud data subsetting and lazy-loading of whole datasets in parallel. These access patterns facilitate the use of big data computational tools that can efficiently process extremely large datasets. Data are provided alongside event-driven notifications. These notifications allow both researchers and engineers to build near-real-time responsive data pipelines to address scaling and facilitate decision support.

This approach to open environmental data has supported breakthroughs in Earth system science; supported industries in economic sectors as diverse as climatetech, renewable energy, reinsurance, and agriculture, to note a few; and driven the development of disaster risk reduction, mitigation, and weather forecasting startups. The potential of NODD data to serve public and private sector is becoming recognized and ramping up. The mission of NODD, and the focus of this paper, is to stimulate more researchers and decision-makers to use these data to analyze, understand, and hopefully address some of the most complex, intractable issues and opportunities of our time. To do so, we first document how NODD works with an eye toward understanding data provenance and usage. Second, we discuss key datasets available via NODD with example use cases. Third, we highlight the multiple ways by which data are accessible via NODD. Last, we place this work in the context of current developments in open science and new initiatives that NODD is developing to further address challenges and improve access to NOAA environmental data.

## RESULTS

### NODD data pipelines

**Fig. 2.**
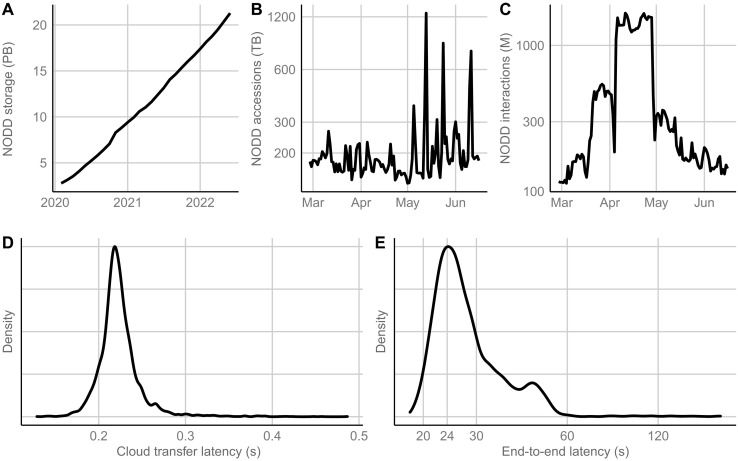
NODD data use across AWS, Azure, and GCP. (**A**) NODD storage growth over time. As of September 2022, NODD makes more than 24 petabytes (PB) of NOAA data available. (**B**) NODD accessions (accessions are listed in terabytes, technically tebibytes, or 2^40^ bytes) per day since March 2022. (**C**) NODD interactions per day since March 2022. Note sustained use in April 2022 exceeding 1.2 billion interactions per day. (**D**) Cloud transfer latency resulting from NODD GOES-16 NetCDF transfer activities for the month of June 2022. This is the additional latency incurred in transferring NOAA data to the cloud. (**E**) End-to-end latency from data generation to public availability on the cloud from NODD GOES-16 NetCDF transfers.

As of September 2022, NODD hosts more than 24 PB (petabytes, technically pebibytes, are equal to 2^50^ bytes) of NOAA environmental data. These data include more than 220 datasets, and more than 200 additional National Marine Fisheries Service (NMFS) datasets will be available as soon as the landing pages are completed (currently in progress). NODD sees sustained access of more than 5 PB of data per month and more than 1 billion interactions per week, with occasional sustained interactions of 1.5 billion per day. Over the past 2 years, NODD has grown from making publicly available a little over 2 PB of NOAA data to hosting more than 24 PB as of September 2022 (Fig. 2A).

This growth has been driven by user demand. NODD sees base-level access of more than 100 TB per day with frequent spikes well above that and exceeding a petabyte a day (Fig. 2B). Access measures direct use of the data and includes both local downloads and use of NOAA data on cloud compute platforms. It measures the amount of data accessed and is reported in terabytes (TB), technically tebibytes, or 2^40^ bytes. NODD also sees a high sustained rate of interactions on a daily basis (Fig. 2C).

Interactions are a combination of all requests to a cloud service provider (or, in this case, all three cloud service providers) and object storage Application Programming Interface (APIs). This includes GET, HEAD, and PUT requests. While some of these requests involve the transfer of data, others involve only the transfer of metadata. High levels of interactions with the data may or may not indicate high levels of access. Programmatic parallel access of metadata can generate high levels of interactions with very low levels of access. Interactions also include internal NODD interactions with the data, which for most datasets on most days is a marginal percentage of interactions. These daily interactions are seldom under 100 million and can exceed 1 billion.

NODD supports extremely performant levels of parallel access to NOAA data. Spikes in accessions and interactions (Fig. 2, B and C) are often not driven by increases in use across multiple datasets (although this can and has occurred). More often, these spikes are due to large-scale use of a single product or products in a single dataset. As a case in point, the AWS GFS dataset saw 1.12 PB of access on 13 May. In another example, for a period of approximately 20 days preceding 28 April, GOES products saw sustained interactions averaging above 1.2 billion/day. This level of access and interaction did not cause any issues with NODD service offerings; there was no throttling, no downtime, no delays, and no interruptions to access.

NODD latency is low. Transfer of NOAA data to the cloud via NODD-managed pipelines tends to add less than a second of latency to overall data availability. As an example, transfer latency of GOES-16 data products via NODD-managed pipelines tends to be between 0.2 and 0.3 s (Fig. 2D). This transfer latency measures the additional amount of time a consumer would have to wait to obtain the data via the cloud versus accessing the data on NOAA’s on-premise system. NODD end-to-end transfer latency, which measures the amount of time it takes from the data to be generated to when the data are publicly available on the cloud and includes NODD cloud transfer latency, is still low (Fig. 2E). As an example, GOES-16 end-to-end transfer latency tends to be around 24 s.

The combination of performant parallel access and low latency creates a new paradigm for public access to open data. Whereas many on-premise systems rely on ordering data with requests for subsets of data taking hours to days or even weeks to fill, NODD data are available all at once, in near real time, with the same format for current and historical data. This level of open access generates opportunities for complete analysis of entire datasets in parallel. Access patterns in combination with anecdotal reports from users suggest that analysis paradigms are shifting focus to access and analysis of complete records from historical holdings through to the present, rather than analysis on subsets of the data.

### NODD key datasets

Making data available via NODD is an intentional, collaborative process. Datasets are prioritized via a combination of stakeholder input, technical considerations, and legal requirements. Cloud service providers, public and private enterprises, data scientists, and researchers often request specific datasets, while NOAA Line Offices and investigators also drive dissemination. With this collaborative approach, a team of engagement experts, cloud engineers, scientists, technical experts, and data consumers work together to design effective and efficient access to key datasets.

**Fig. 3.**
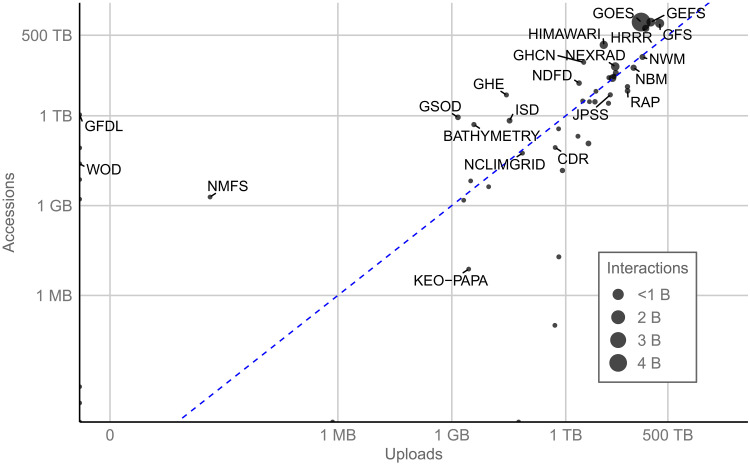
Select NOAA datasets made publicly available on the cloud via NODD. Points denote median monthly accessions (*y* axis), uploads (*x* axis), and interactions (size of points) for the past 5 months across AWS, Azure, and GCP. Dashed blue line is a one-to-one ratio between accessions and uploads. Datasets above the blue line indicate more accessions than uploads.

NODD hosts a number of key NOAA datasets on the public cloud that are both heavily used and frequently updated (Fig. 3). This heavy use can be independent of the overall size of the dataset. For example, the NOAA GFS represents 2.7 PB of holdings across AWS, Azure, and GCP with a monthly accessions-to-uploads ratio of 7.6. This means roughly that, on average, for every byte of data uploaded, that byte is accessed 7.6 times. On the other hand, the Global Historical Climatology Network (GHCN) dataset represents only 112 GB of holdings across AWS and Azure with a monthly accessions-to-uploads ratio of 18.5. This means roughly that, on average, for every byte of GHCN data uploaded, that byte is accessed 18.5 times.

A number of these key datasets stand out in terms of their size, their accession patterns, the amount of interactions, or some combination of these factors. To that end, a number of datasets that are a combination of being particularly large, highly accessed, and supporting a high level of interactions are highlighted below. A full listing of NODD datasets is available on the NODD homepage (https://noaa.gov/nodd).

### Satellite

NODD makes available a wide swath of environmental satellite information both in near real time and historically.

#### Geostationary Operational Environmental Satellite

The GOES satellite program is a collaboration between NOAA and NASA. GOES-16 and GOES-17 data are currently available through NODD across AWS, Azure, and GCP, including near-real-time feeds and period of record historical data. GOES-18 provisional data were made publicly available in late 2022. These data include continuous weather imagery and sensor data useful for monitoring and predicting extreme weather events (*2*). As of July 2022, GOES-16 and GOES-17 data represent more than 4.7 PB of NODD holdings and see some of the highest overall levels of interactions and accessions.

#### Joint Polar Satellite System

The Joint Polar Satellite System (JPSS) program is also a collaboration between NOAA and NASA. JPSS data were onboarded to NODD in 2022 and are currently available on AWS. JPSS holdings will be expanding over the coming year to the other cloud service providers and include imagery and monitoring products that address every aspect of the Earth system (*3*).

#### Himawari-8/9

The Himawari satellites are owned and operated by the Japan Meteorological Agency (JMA), and data are made available through a collaboration with NOAA and AWS. The data include imagery for monitoring and forecasting of east Asia and the western Pacific (*4*). Himawari-8 historical and near-real-time data are available via NODD. NODD onboarded Himawari-9 in late 2022 with holdings expanding over the coming year.

### Radar

#### Next Generation Weather Radar

Next Generation Weather Radar (NEXRAD) weather radar data are produced by 160 high-resolution, S-band Doppler weather radars through a joint effort of the National Weather Service (NWS), Federal Aviation Administration (FAA), and U.S. Air Force (*5*). NODD makes near-real-time NEXRAD level II and level III data available via GCP. NEXRAD data are also available on AWS, though managed by Unidata.

### Weather

Weather models and forecast data are provided via NODD across all three cloud service providers. The GFS, Global Ensemble Forecast System (GEFS), and High-Resolution Rapid Refresh (HRRR) datasets together comprise more than 7 PB of NODD holdings.

### Model

#### Global Forecast System

The GFS is a comprehensive global weather forecast model produced by the National Centers for Environmental Prediction (NCEP). This model is available operationally gridded at 0.25° and 0.5° and is made available four times per day for running forecasts at the 00Z, 06Z, 12Z, and 18Z cycles. Event-driven real-time notifications are available.

#### Global Ensemble Forecast System

The GEFS is a weather model from NCEP that generates a suite of 21 forecasts used as ensemble members to facilitate examination of uncertainty in weather forecasts (*6*). Like GFS, GEFS is available four times daily but is gridded at 1°. Event-driven real-time notifications are also available.

#### High-Resolution Rapid Refresh

The HRRR model is an atmospheric model updated hourly and gridded at 3 km across the contiguous United States, Alaska, and Hawaii (*7*, *8*). It assimilates radar data every 15 min and includes metrics as varied as inline smoke prediction and lake temperatures. Event-driven real-time notifications are also available.

### Observation

#### Global Historical Climatology Network

The GHCN data are available in both monthly and daily formats (*9*, *10*). Hourly GHCN data are expected to be available by the end of 2023. The network data contain climate summaries from more than 100,000 land surface stations globally (including 180 countries). These data undergo a rigorous and common quality assurance process and are updated daily.

#### NOAA U.S. Climate Gridded Dataset

The U.S. Climate Gridded Dataset is a 5-km gridded version of four primary variables from the GHCN daily dataset for the continental United States (*11*, *12*). Preliminary data are available weekly, while scaled data are available monthly. State and county summaries of these gridded data are also available.

### Ocean

#### World Ocean Database

The World Ocean Database is a collection of historical subsurface ocean profile information, including temperature, salinity, oxygen, and nutrients, among others (*13*). It is compiled using a variety of sources, including the Argo program, and updated quarterly.

#### Bathymetry

High-resolution bathymetry data from NOAA and the U.S. Army Corps of Engineers are available from sources ranging from hydrographic surveys and bathymetric lidar.

### Period of record

The NODD program makes a suite of climate data records (CDRs) available across all three cloud service providers. These CDRs are created by NOAA National Centers for Environmental Information (NCEI) as extensively vetted longitudinal assessments of changes in the land, atmosphere, oceans, and ice sheets. NCEI maintains and updates CDRs using standards from the National Research Council.

### NODD data access

Access to NOAA data through NODD object storage on AWS, Azure, and GCP is provided at no cost to the user. Users do not have to pay to access data, pay for any egress of data, and authenticate to access data, nor have cloud accounts to use the data. The infrastructure that each cloud service provider has put behind their object storage platforms makes the data available simultaneously in parallel with no throttling: Each platform supports several thousand requests per second. This performant access is what enables single users to access petabytes of data in a single day. Each cloud service provider also offers free compute services, which require registration but not a cloud account. Examples include Microsoft Planetary Computer, Google Colaboratory, Google Earth Engine, and AWS Sagemaker Studio Lab.

**Fig. 4.**
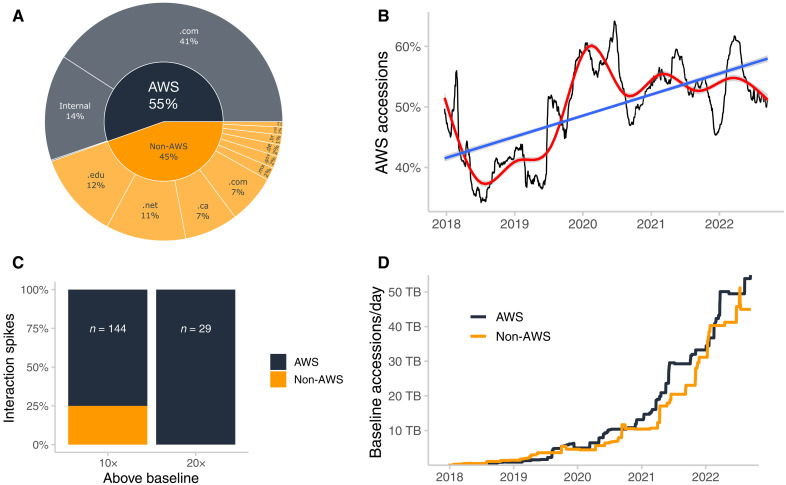
Patterns of cloud access of NODD data. (**A**) Interactions from top-level domains for the month of June 2022 for NODD data on AWS. (**B**) Rolling 90-day median of NODD accessions on AWS by AWS resources. Red line denotes a generalized additive model using smoothing cubic splines, while the blue line denotes a linear model. (**C**) Spikes in interactions above a baseline rolling 90-day minimum. (**D**) Baseline (rolling 90-day minimum) accessions of NODD AWS holdings by AWS and non-AWS resources. AWS versus non-AWS resource use is distinguished by the IP address of the specific request. Requests originating from IP addresses owned by AWS are labeled AWS. All others are labeled non-AWS.

NODD has seen increasing within- and cross-cloud access over time. As an example, for June 2022, a little more than half of interactions were from within AWS [AWS-owned and AWS private internet providers (IPs)] for NODD data on AWS (Fig. 4A). Slightly less than half were from the public internet, and almost 12% came from .edu top-level domains (Fig. 4A). For NODD data holdings on AWS, the percentage of accessions originating from AWS-owned IP address is trending upward over time with seasonal and yearly periodicity (Fig. 4B). In particular, heavy use of NODD holdings on AWS as characterized by spikes 10× or 20× greater than baseline is driven by on-cloud resources (Fig. 4C). Baseline accessions of NODD data holdings on AWS also tend to be greater from AWS resources (Fig. 4D).

### Data availability

#### Amazon Web Services

AWS facilitates dataset discovery and access through the AWS Registry of Open Data Program (RODA) and through the AWS Data Exchange (ADX). In addition to provenance information, access links to AWS object storage and event-driven notifications are available. Examples demonstrating use of NODD datasets are also available through the Registry of Open Data dataset homepages. NODD presented at AWS re:Invent 2022 with the presentation available in (*14*).

#### Microsoft Azure

Microsoft Azure facilitates discovery and access to NODD datasets via its Planetary Computer, which offers a SpatioTemporal Asset Catalog (STAC) API and compute resources for analysis. Data are also available through Azure-based object storage. Examples of using NODD data via the Planetary Computer are also provided.

#### Google Cloud Platform

NODD data can be discovered and accessed through the Google Cloud Marketplace, which points to data available through Google Object Storage. NODD data can also be accessed through Google Earth Engine, which pairs data access via an API with available compute resources. Examples of using NODD data with Google Earth Engine are also provided.

## DISCUSSION

The accessibility of open environmental data via NODD is a foundation for petabyte-scale Earth system science. NODD was designed with Findability, Accessibility, Interoperability, and Reusability (FAIR) principles in mind to further data access and reusability of NOAA data (*15*). On NODD, NOAA data are findable through the searchable interfaces of each cloud service provider, which allow for discovery using metadata in both human-readable and machine-readable formats. NODD data are accessible through free, open, and universally implementable protocols both at the storage layer and through additional cloud service provider offerings (Fig. 1). NODD enhances interoperability through use of common formats and identifiers. NODD drives reuse of data through clear provenance and implementation of NOAA data and metadata standards.

The open, performant access to data provided through NODD using FAIR principles is already leading to new insights in our understanding of the Earth system. For example, open cloud–based access to NOAA data has been used to quantify bird declines across the continental United States (*16*, *17*), identify insect population surges in California farmland, predict flood risk at a granular level (*18*, *19*), provide insights into wind speed prediction analytics for energy demand and forecasting, and drive improvements in wildfire detection and monitoring using near-real-time data, as well as support business and industries in their environmental, social, and governance evaluation and metrics.

NODD continues to expand its ability to facilitate access to petabyte-scale Earth system data in the cloud and is investing heavily in new initiatives to support broader, more performant access. NODD continues to onboard additional NOAA datasets, including datasets from the National Marine Fisheries Service, the Multi-Radar Multi-Sensor operational dataset from the National Severe Storms Laboratory, and the Real-Time Mesoscale Analysis/Unrestricted Mesoscale Analysis from NCEP. Addition of new datasets is driven by users, cloud service providers, and stakeholder requests as well as from NOAA Line Offices. NODD welcomes additional interest and feedback from its user and partner communities regarding interest in additional data on the cloud.

In addition, NODD is making key investments to support performant access to NOAA data through the use of data formats that further enhance scaling Earth system analysis. Research efforts are underway to explore and implement performance benefits from the Apache Parquet, Zarr, and Cloud Optimized GeoTIFF file formats. In particular, NODD works on connecting communities such as Pangeo to NOAA data (*20*). These ongoing efforts are related to building robust conversion pipelines that preserve the appropriate metadata and include appropriate documentation of provenance.

NODD is furthering its efforts in providing near-real-time data in a streaming format, which would both reduce latencies and allow for application of in-stream machine learning pipelines for rapid prediction. Requests for streaming access have focused on satellite data products that support analysis in extreme weather events, wildfires, flooding, and other natural disasters. This is an area of active exploration where advances and public streaming data offerings could open additional branches of real-time and near-real-time predictive Earth system science.

The NODD vision is to accelerate open science by making all of NOAA public data freely available in the cloud. A key aspect of this vision is addressing challenges in accessibility. The structure and format of data, even when it is hosted on the cloud, can prevent efficient analysis or preclude analysis altogether. NODD is actively engaged in building systems that improve accessibility through more performant formats and data structures. NODD also invests heavily to decrease the latency of data delivery and collaborates with cloud service providers to monitor and enhance their storage and tool development.

NODD emphasizes the fair and discoverable nature of environmental data to enhance tool development and support artificial intelligence/machine learning (AI/ML). NODD is supporting efforts to build and enhance its data governance activities and policies so it can meet the future needs of technical users who are expecting AI/ML-ready environmental data. NODD’s current open data holdings are a first step along a path that provides access to NOAA resources in the cloud.

## MATERIALS AND METHODS

### NODD pipelines

NODD relies on a number of sources within NOAA to make data available on the cloud service providers (Fig. 1). In many cases, data originate at the instrumentation level on satellites, in situ environmental sensors, or via physical collections of information (images, video, genomes, etc.). In other cases, data origins lie in model runs on NOAA internal compute capacity. The collection, collation, archiving, and dissemination of this information through NOAA systems are managed internally at NOAA. Cloud service providers can request specific datasets, and NOAA can also place specific datasets on its allocation. While there are overlaps in the data holdings across the cloud service providers, some datasets are only available through specific providers.

NOAA data are accessed by NODD via the Weather and Climate Operational Supercomputing System (WCOSS), Comprehensive Large Array Data Stewardship System (CLASS), Production Distribution and Access (PDA), Research and Development HPC System (RDHPCS), and NCEP Production FTP Server (FTPPRD) HPC systems. These systems are constantly monitored for any updates, and new data are transferred to cloud holdings on all three cloud service providers using highly resilient, performant, and fault-tolerant Apache NiFi deployments. As part of this process, data provenance is closely tracked and data are validated using checksums. The architecture of this system is such that the pipeline for each dataset is constructed with multiple alternative sources and failovers. Outages upstream of NODD are quickly and effectively mitigated via this architecture. Because these pipelines often source from multiple locations, NODD has experienced extremely reliable uptime maintaining data feeds even when some source systems go down.

In addition to NODD-managed pipelines that directly access NOAA computing systems, NODD also works closely with each NOAA Line Office and with dataset principal investigators to push their data directly into the cloud using the cloud native tooling of each cloud service provider. These pipelines are managed by individual dataset teams supported by NODD and can come from NOAA research laboratories under the purview of NOAA Oceanic and Atmospheric Research like the Geophysical Fluid Dynamics Laboratory, Earth Prediction Innovation Center, and the Atlantic Oceanographic and Meteorological Laboratory.

NODD data in the cloud are initially and principally held in object storage on the cloud: Simple Storage Service (S3) on AWS, Azure Blob Storage (ABS) on Microsoft Azure, and Google Cloud Storage (GCS) on GCP. Object storage serves as a base layer of performant public access to NOAA environmental data. From this base layer, a number of other features are offered on each cloud service platform, including event notifications to support event-driven workflows, integration with cloud compute services, and inclusion in platforms like Google Earth Engine and Microsoft Planetary Computer.

In addition, NODD works closely with cloud service providers, partners, and other stakeholders to optimize cloud holdings for improved accessibility and interoperability connections with other datasets. This work involves format conversions and integration into catalog-based APIs. Format conversions to more accessible cloud-native formats like Cloud Optimized Geo-Tiffs, Zarr, and Parquet are done using event-driven serverless pipelines. Connections and integrations are achieved principally through development and publication of STAC APIs, which enable structured API requests to specific subsets of available data.

### NODD data access

A principal component of effective and efficient data access is dataset discovery. To that end, NODD integrates not only with the open data platforms from each cloud service provider but also with NOAA’s internal data catalogs and listings to facilitate dissemination and discovery of NOAA cloud data. In addition, NODD works closely with researchers and other stakeholders to include examples of using these data on the cloud through data documentation, notebooks, tutorials, webinars, and workshops.

A second component of effective and efficient data access involves multiple methods of access. NODD has intentionally designed the platforms to be accessible by a broad and diverse community. The base levels of NODD holdings in cloud storage are accessible both programmatically and via interactive web-based explorers to assist users across the technical spectrum.

In addition to providing effective and efficient data access to a diverse community, NODD also focuses on developing solutions that increase and instill institutional equity for those with difficulty accessing and using NOAA data. NODD is working to lower and/or remove barriers of entry to data access by supporting free anonymous access, connecting users with subject matter experts, and developing introductory guides to usage of key datasets. It also involves working with each cloud service provider to provide free computing time and training to work with these data. Simply having NOAA data freely available on the cloud does not solve data equity issues, however. Open and free data access alongside training and compute offerings that are free are necessary but not sufficient steps on the pathway to data equity.

## Supplementary Materials

### This PDF file includes:

Acronyms
