# Supplementary material for: NOAA Open Data Dissemination: Petabyte-scale Earth system data in the cloud
Source: Sci Adv. 2023 Sep 20;9(38):eadh0032. doi: 10.1126/sciadv.adh0032 (PMC10511182; doi:10.1126/sciadv.adh0032)
Supplement: Supplementary file 1 — Acronyms [file sciadv.adh0032_sm.pdf]

Science Advances
AAAS

Supplementary Materials for

NOAA Open Data Dissemination: Petabyte-scale Earth system data in the cloud

Denis S. Willett *et al.*

Corresponding author: Denis S. Willett, denis_willett@ncsu.edu

Sci. Adv. **9**, eadh0032 (2023)
DOI: 10.1126/sciadv.adh0032

This PDF file includes:

Acronyms

ABS	Azure Blob Storage, Azure Object Storage
AWS	Amazon Web Services
CDR	Climate Data Record
CLASS	Comprehensive Large Array Data Stewardship System
FTPPRD	NCEP Production FTP Server
GCP	Google Cloud Platform
GEFS	Global Ensemble Forecast System
GFDL	Geophysical Fluid Dynamics Laboratory
GFS	Global Forecast System
GHCN	Global Historical Climatology Network
GHE	Global Hydro Estimator
GOES	Geostationary Operational Environmental Satellite
GSOD	Global Surface Summary of the Day
HRRR	High Resolution Rapid Refresh
ISD	Integrated Surface Daily
JPSS	Joint Polar Satellite System
NBM	National Blend of Models
NCEP	National Centers for Environmental Prediction
NCLIMGRID	U.S. Gridded Climate Dataset
NDFD	National Digital Forecast Database
NESDIS	National Environmental Satellite, Data, and Information Service
NEXRAD	Next Generation Weather Radar
NMFS	National Marine Fisheries Service
NODD	NOAA Open Data Dissemination
NWM	National Water Model
NWS	National Weather Service
PDA	Production Distribution and Access
RDHPCS	Research and Development HPC System
S3	Simple Storage Service, AWS Object Storage
STAC	SpatioTemporal Asset Catalog
WCOSS	Weather and Climate Operational Supercomputing System
WOD	World Ocean Database
